# Single-agent interleukin-2 in the treatment of metastatic melanoma

**DOI:** 10.3747/co.2007.97

**Published:** 2007-02

**Authors:** T. Petrella, I. Quirt, S. Verma, A.E. Haynes, M. Charette, K. Bak, the members of the Melanoma Disease Site Group of Cancer Care Ontario’s Program in Evidence-Based Care

**Affiliations:** * Sunnybrook Regional Cancer Centre, Toronto, Ontario; † Princess Margaret Hospital, Toronto, Ontario.; ‡ The Ottawa Hospital, Ottawa, Ontario.; § Cancer Care Ontario’s Program in Evidence-Based Care, McMaster University, Hamilton, Ontario; || Please see the Web site of the Cancer Care Ontario Program in Evidence-Based Care (www.cancercare.on.ca/index_AboutthePEBC.htm#dsg) for a complete list of current members of the Melanoma Disease Site Group.

**Keywords:** Melanoma, interleukin-2, il-2, practice guideline

## Abstract

**Questions:**

What is the role of single-agent interleukin-2 (il-2) in the treatment of adults with metastatic melanoma?

If there is a role for single-agent il-2, what patient population can appropriately be considered for treatment?

If there is a role for single-agent il-2, what dose and schedule are appropriate?

What are the toxicities associated with il-2?

**Perspectives:**

Many agents have been investigated for antitumour activity in melanoma, but few have shown promising response rates. Early detection, appropriate surgery, and adjuvant therapy have all improved outcomes, but approximately one third of patients with early-stage disease will nevertheless develop metastases. Single-agent il-2 has attracted much attention over the past several years. A number of randomized trials and many phase ii trials investigating single-agent il-2 suggest that this systemic treatment produces durable responses in melanoma patients.

Given the dismal survival of patients with meta-static melanoma and the limited availability of effective treatments, the Melanoma Disease Site Group (dsg) of Cancer Care Ontario’s Program in Evidence-Based Care (pebc) felt that the durable responses seen with il-2 treatment warranted closer examination.

**Outcomes:**

Primary outcomes of interest included objective response rate, complete response rate, duration of response, toxicity, and quality of life. Secondary outcomes of interest included progression-free survival and overall survival.

**Methodology:**

A systematic review was developed, and clinical recommendations relevant to patients in Ontario were drafted. The practice guideline report was reviewed and approved by the Melanoma dsg, which comprises medical oncologists, surgeons, and dermatologists. External review by Ontario practitioners was obtained through a mailed survey, the results of which were incorporated into the practice guideline. Final review and approval of the practice guideline was obtained from the pebc’s Report Approval Panel.

**Results:**

The present practice guideline reflects the integration of the draft recommendations based on a systematic review of the available evidence with the feedback obtained from external review by practitioners and the Report Approval Panel.

**Practice Guideline:**

No studies have compared il-2 to the current standard of care—dacarbazine (dtic)—or to placebo in the treatment of metastatic melanoma. After reviewing and weighing the evidence that does exist, the opinion of the Melanoma dsg is that high-dose il-2 is a reasonable treatment option for a select group of patients with metastatic melanoma:

- Patients should have a good performance status (Eastern Cooperative Oncology Group 0–1) and a normal lactate dehydrogenase level.
- Patients should have fewer than three organs involved or have cutaneous and/or subcutaneous metastases only, and no evidence of central nervous system metastases should be present.

In this select group of patients, il-2 treatment can produce durable complete remissions.

High-dose il-2 is recommended to be given at 600,000 IU/kg per dose, delivered intravenously over 15 minutes, every 8 hours, for a maximum of 14 doses. High-dose il-2 delivery is recommended to be done in a tertiary-care facility by staff trained in the provision of this treatment and with appropriate monitoring. To facilitate treatment and to develop expertise in this therapeutic modality, the dsg recommends that high-dose il-2 programs be established in one or two centres in Ontario.

**Qualifying Statements:**

High-dose il-2 has response rates that are similar to those seen with standard chemotherapy. However, unlike chemotherapy, il-2 demonstrates low but durable complete response rates that may lead to years of benefit for patients with metastatic melanoma.

Based on the available data assessing prognostic factors and patient selection, patients with non-visceral metastases and fewer metastatic sites have a much higher response rate. In these select patients, high dose il-2 may be considered for first-line therapy.

The lack of large randomized trials comparing il-2 to dtic or other chemotherapy means that recommendations for this guideline are based largely on phase ii data and limited phase iii data. Further randomized data will not soon become available, because no randomized trials are currently ongoing or planned. Interleukin-2 is currently widely used in the United States, and it is an approved therapy in both Canada and the United States.

## 1. QUESTION

What is the role of single-agent interleukin-2 (il-2) in the treatment of adults with metastatic melanoma? Primary outcomes of interest included objective response rate, complete response (cr) rate, duration of response, toxicity, and quality of life (qol). Secondary outcomes of interest included progression-free survival and overall survival.

If there is a role for single-agent il-2, what patient population can appropriately be considered for treatment?

If there is a role for single-agent il-2, what dose and schedule are appropriate?

What are the toxicities associated with il-2?

## 2. CHOICE OF TOPIC AND RATIONALE

Like just a few other malignancies, malignant melanoma continues to increase in both incidence and mortality. In 2005 in Canada, approximately 4400 new melanoma cases were expected to be diagnosed, and 880 patients to die from the disease 1. Part of this increase can be attributed to more frequent diagnosis of earlier-stage lesions, but diagnosis cannot be the sole contributing factor, because mortality rates for melanoma are also increasing. The increasing mortality suggests that new methods are required to treat the disease, either to prevent recurrence or when surgery is no longer feasible.

Despite low response rates, rare durable responses, and lack of a survival benefit, dacarbazine (dtic) is currently considered to be the standard treatment for metastatic melanoma 2–4.

Given the dismal survival rate for patients with metastatic melanoma and the limited availability of effective treatments, the Melanoma dsg felt that il-2 treatment warranted closer examination.

## 3. METHODS

### 3.1 Guideline Development

The present practice guideline report was developed by the Melanoma dsg of Cancer Care Ontario’s Program in Evidence-Based Care (pebc), using the methods of the practice guidelines development cycle 5. It is a convenient and up-to-date source of the best available evidence on il-2 treatment for patients with metastatic melanoma, developed through systematic review, evidence synthesis, and input from practitioners in Ontario. The systematic review that forms the basis of this report (and that is currently under consideration for publication elsewhere) was used by the Melanoma dsg to formulate draft recommendations promoting evidence-based practice in Ontario.

External review for the practice guideline report was obtained through a mailed survey of Ontario practitioners. The survey consisted of items that addressed the quality of the draft practice guideline report and asked whether the recommendations should serve as a practice guideline. The efficacy of the external review process has been described elsewhere 6. Final approval of the original practice guideline report was obtained from the pebc’s Report Approval Panel. All members of the Melanoma dsg disclosed information on potential conflicts of interest. No conflicts were declared.

### 3.2 Literature Search Strategy

The medline, embase, and Cochrane Library databases (1985 to 2006) were systematically searched for eligible randomized phase iii and ii trials. Abstracts published in the proceedings of the 1997–2005 annual meetings of the American Society of Clinical Oncology were also systematically searched for evidence relevant to the present practice guideline report.

### 3.3 Results

One systematic review 7, five randomized trials 8–12 that compared single-agent il-2 with il-2 combination therapy, and twelve single-arm phase ii trials 13–25 (one of which was reported in two separate publications 15,16) were eligible for inclusion in this systematic review of the evidence. In addition, the literature search identified a qol report 24 for patients included in one of the randomized trials.

None of the randomized controlled trials (rcts) compared single-agent il-2 to standard therapy or to placebo. In addition, the rcts included varying regimens and doses of il-2 and combined il-2 with various agents (lymphokine-activated killer cells, interferon, and histamine dihydrochloride). Because of this heterogeneity in the rcts, the Melanoma dsg decided against performing a meta-analysis of the results.

Data from three rcts 8–10 demonstrated that single-agent il-2, when given in high doses, can elicit an objective response rate of 5%–27%, with a cr in 0%–4% of patients. In addition, patients who achieved a cr in the five randomized trials also demonstrated consistent long-term response rates that ranged from 6 months to 66+ months (median: 27 months). Similarly, the non-comparative phase ii trials of high-dose single-agent il-2^13^–^15^ consistently reported objective response rates of 10%–33% with a cr rate ranging from 0% to 15%. Complete responders in these trials also demonstrated impressive long-term responses that ranged from 1.5 months to 148 months (median: 70 months).

The gathered data show that carefully selected patients have the highest chance of response. Patients with a good performance status [Eastern Cooperative Oncology Group (ecog) 0–1], a normal lactate dehydrogenase (ldh) level, and fewer than three organs involved or cutaneous and/or subcutaneous metastases only have the highest probability of responding and achieving a durable cr. This carefully selected group of patients should be considered for treatment with high-dose il-2.

## 4. DSG CONSENSUS PROCESS

The draft guideline was circulated for review and discussion by the Melanoma dsg on June 21, 2005. Most of the members conceded that, given the available data, it would be optimal to use il-2 as first-line therapy in a select group of patients when disease burden is at its lowest. However, it should be noted that one member of the Melanoma dsg was not comfortable with the recommendations set out in this document, stating that il-2 “is and remains an investigational drug” and thus should not be used as standard therapy.

## 5. PRACTITIONER FEEDBACK

Recommendations in the form of a draft practice guideline report and systematic review were prepared by the Melanoma dsg and circulated to Ontario practitioners.

### 5.1 Methods

Feedback was obtained through a mailed survey of 176 practitioners in Ontario (medical oncologists, radiation oncologists, and surgeons). The survey consisted of items evaluating the methods, results, and interpretive summary used to inform the recommendations and whether the recommendations should be approved as a practice guideline. Written comments were invited. The survey was mailed July 8, 2005. Follow-up reminders were sent at 2 weeks (post card) and 4 weeks (complete package mailed again). The Melanoma dsg reviewed the results of the survey.

### 5.2 Results

Of the 176 surveys mailed, 65 responses were received (37% response rate). Responses included returned completed surveys plus telephone, fax, and e-mail responses. Of the practitioners who responded, 29 (45%) indicated that the report was relevant to their clinical practice, and they completed the survey. Most of the practitioners surveyed (93%) indicated that a practice guideline on this topic was needed. All the respondents agreed that the draft recommendations in the report are clear, and 90% agreed with the recommendations as stated. Most respondents (82%) agreed that the report should be approved as a practice guideline.

### 5.3 Summary of Written Comments

Twenty-two respondents (76%) provided written comments. These were the main points contained in the written comments:

- Two practitioners felt that the evidence was insufficient to recommend the use of il-2 over other, less toxic drugs. One of these two practitioners stated that the evidence of benefit is overstated and noted that, in several studies, the toxic death rate approximates cr rates. This practitioner pointed out that the guideline does not indicate “how to select eligible patients or what evidence supports selection (i.e., no prospective studies showing selected patients do better).” A third practitioner also agreed that only a limited group would benefit and that it would be difficult to determine those fit enough to withstand the treatment.
- Six respondents provided favourable comments and supported the guideline recommendations. Two of those six practitioners felt that administering il-2 in Ontario is a more economically feasible option than is sending patients to the United States. One respondent felt that treating complete responders would be practical; however, the challenge in establishing wait lists for those patients and centres in which to treat them, together with other associated financial costs, would limit the application of the guidelines. It was also suggested that issues such as the cost of medicine, hospital visits, and treatment of complications should be addressed.
- Nine practitioners stated that they do not treat melanoma or that the guideline does not apply to them.
- Six practitioners provided suggestions for future topics.

### 5.4 Modifications/Actions

The Melanoma dsg discussed the comments resulting from practitioner feedback and responded as follows:

- The dsg acknowledged that high-quality evidence suggesting a clear survival benefit for il-2 therapy is currently lacking. However, given the dismal survival of patients with metastatic melanoma and the fact that no other therapy offers the possibility of a durable complete remission, the dsg believes that recommending the use of high-dose il-2 to a select group of patients is reasonable. The specific patient eligibility for this treatment is defined in the document.
- The dsg also agreed that high-dose il-2 therapy presents considerable grades 3 and 4 toxicity. However; these adverse effects have become increasingly manageable, and they resolve upon termination of treatment. Delivery of the treatment by adequately trained professionals in a designated tertiary care facility and following the National Cancer Institute’s guidelines for safe administration will ensure safety and proper patient monitoring.
- The Melanoma dsg agreed that administering il-2 therapy in Ontario, rather than referring patients to the United States for this treatment, is a more economically feasible option. Creating high-dose il-2 programs in one or two centres in Ontario will not only facilitate patient treatment, but will also develop expertise in this therapeutic modality. The dsg recognized the importance of the financial concerns raised by some of the practitioner respondents; however, the dsg’s main obligation is to establish clinical effect and determine best evidence-based practice. Thus, assessing issues of cost and economic benefit is outside of the purview of this document.

The dsg therefore decided to make no changes to the draft guideline.

## 6. REPORT APPROVAL PANEL

The final evidence-based report was reviewed and approved by the pebc’s Report Approval Panel in May 2006. The Panel consists of two members, including an oncologist with expertise in clinical and methodology issues.

### 6.1 Results

Key issues raised by the pebc Report Approval Panel included these:

- The rcts address all of the relevant outcomes, although the data from the phase ii trials are more favourable. Inclusion of the phase ii data could be viewed as biased; clearer justification for the inclusion of the phase ii data should therefore be provided.
- Further discussion of the trade-offs of treatment would be useful. The treatment may offer the best palliation option; however, it can lead to a miserable death with adverse effects for a slim chance of survival with unknown qol because no appropriate comparisons are available.
- It appears that access to this treatment is a topical subject, although the relevant background becomes apparent only in the section on practitioner feedback. If access to therapy was a factor that contributed to the dsg’s selection of this topic, it would be appropriate to state this fact in the introduction.
- The recommendations are based principally on studies that provide a lower level of evidence and that assess an outcome that does not usually drive policy decisions. The dsg has done a relatively good job of explaining how it concluded that a recommendation to support availability of this therapy was appropriate. However, given the relatively unusual nature of recommending a therapy based on such data, and given the toxicity of the agent (in that special sites of provincial expertise may have to be developed), the dsg needs to go further in addressing how the potential benefits of this therapy “outweigh” the limitations of the data. Specifically:
  - Context regarding the importance of response as an outcome measure would be helpful.
  - An indication of whether the prolonged periods of disease control seen in patients with a more complete response is unique or whether responses of this kind are seen in patients who respond to other therapies would be helpful. There is a further risk of criticism that the dsg has compared overall responses to il-2 with the historical results associated with other therapies, but is providing only the data for durable responses with il-2.
  - The dsg also risks criticism for considering il-2 as first-line therapy, given that this consideration appears to be based principally on analyses of prognostic factors. The difficulty is that patients with earlier-stage disease will always do better than those with more advanced disease, regardless of the therapy provided. More background is required to support this supposition.
- The dsg also should consider providing broader context for the Agarwala study. This rct was the largest of those reported, and it described differences in median time to disease progression (likely a more “policy–determining” outcome than response) and median quality-adjusted survival. Although the potential limitations of these data can be appreciated, an explanation of the reasons for not using the results of this trial to form recommendations when trials that have more severe limitations have been used would be helpful.

### 6.2 Modifications/Actions

The Melanoma dsg discussed the comments resulting from the pebc Report Approval Panel and responded with these comments and modifications:

- The dsg felt that the inclusion of phase ii data was necessary because of the limited availability of phase iii data. To further justify the inclusion of phase ii data, a Qualifying Statement was added to the document.
- The dsg points out that il-2 is not a palliative treatment. The response rates offered by il-2 are similar to those offered by the current standard of care, dtic; adverse events are manageable and reversible; and death attributable to treatment is no longer common.
- Access to therapy was not a contributing factor in the dsg’s selection of this topic. The purpose of the present document is to establish clinical effect and to determine best evidence-based practice. However, the dsg acknowledges that access to treatment is addressed in the document.
- The discussion section of the systematic review was expanded to further address the benefits of il-2 therapy, and Qualifying Statements were added to explain the dsg’s decision to recommend il-2 as first line therapy.
- The dsg decided not to use the results of the Agarwala trial to form its recommendations because that trial randomized its patients to treatment with low-dose il-2. Low-dose il-2 has consistently lower overall response rates (2%–5%) than does high-dose il-2, and hence low-dose treatment was not part of the recommendation.

## 7. PRACTICE GUIDELINE

The present practice guideline reflects the integration of the draft recommendations with feedback obtained from the external review process. The guideline has been approved by the Melanoma dsg and the pebc’s Report Approval Panel.

### 7.1 Recommendations

No studies have compared il-2 to the current standard of care—dacarbazine (dtic)—or to placebo in the treatment of metastatic melanoma. After reviewing and weighing the evidence that does exist, the opinion of the Melanoma dsg is that high-dose il-2 is a reasonable treatment option for a select group of patients with metastatic melanoma:

- Patients should have a good performance status (ecog 0–1), and a normal ldh level.
- Patients should have fewer than three organs involved or have cutaneous and/or subcutaneous metastases only, and they should have no evidence of central nervous system metastases.

In this select group of patients, il-2 treatment can produce durable complete remissions.

High-dose il-2 is recommended to be given at 600,000 IU/kg per dose, delivered intravenously over 15 minutes, every 8 hours, for a maximum of 14 doses. High-dose il-2 delivery is recommended to be done in a tertiary-care facility by staff trained in the provision of this treatment and with appropriate monitoring. To facilitate treatment and develop expertise in this therapeutic modality, the dsg recommends that high-dose il-2 programs be established in one or two centres in Ontario.

### 7.1 Qualifying Statements

High dose il-2 has response rates that are similar to those seen with standard chemotherapy. However, unlike chemotherapy, il-2 demonstrates low but durable complete response rates that may lead to years of benefit for patients with metastatic melanoma.

Based on the available data assessing prognostic factors and patient selection, patients with non-visceral metastases and fewer metastatic sites have a much higher response rate. In these select patients, high-dose il-2 may be considered for first-line therapy.

The lack of large randomized trials comparing il-2 to dtic or other chemotherapy means that recommendations for this guideline are based largely on phase ii data and limited phase iii data. Further randomized data will not soon become available, because no randomized trials are currently ongoing or planned. Interleukin-2 is currently widely used in the United States, and it is an approved therapy in both Canada and the United States.

## 8. PRACTICE GUIDELINE DATE

This clinical practice guideline report is based on work completed in May 2006. All approved pebc clinical practice guideline reports are updated regularly. Please see the pebc Web site, www.cancercare.on.ca/index_AboutthePEBC.htm, for a complete list of current and ongoing projects.
